# Improving the use of research evidence in guideline development: 2. Priority setting

**DOI:** 10.1186/1478-4505-4-14

**Published:** 2006-11-29

**Authors:** Andrew D Oxman, Holger J Schünemann, Atle Fretheim

**Affiliations:** 1 Norwegian Knowledge Centre for the Health Services, P.O. Box 7004, St. Olavs plass, N-0130 Oslo, Norway; 2 INFORMA, S.C. Epidemiologia, Istitituto Regina Elena, Via Elio Chianesi 53, 00144 Rome, Italy; 3 Norwegian Knowledge Centre for the Health Services, P.O. Box 7004, St. Olavs plass, N-0130 Oslo, Norway

## Abstract

**Background:**

The World Health Organization (WHO), like many other organisations around the world, has recognised the need to use more rigorous processes to ensure that health care recommendations are informed by the best available research evidence. This is the second of a series of 16 reviews that have been prepared as background for advice from the WHO Advisory Committee on Health Research to WHO on how to achieve this.

**Objectives:**

We reviewed the literature on priority setting for health care guidelines, recommendations and technology assessments.

**Methods:**

We searched PubMed and three databases of methodological studies for existing systematic reviews and relevant methodological research. We did not conduct systematic reviews ourselves. Our conclusions are based on the available evidence, consideration of what WHO and other organisations are doing and logical arguments.

**Key questions and answers:**

There is little empirical evidence to guide the choice of criteria and processes for establishing priorities, but there are broad similarities in the criteria that are used by various organisations and practical arguments for setting priorities explicitly rather than implicitly,

**What criteria should be used to establish priorities?:**

• WHO has limited resources and capacity to develop recommendations. It should use these resources where it has the greatest chance of improving health, equity, and efficient use of healthcare resources.

• We suggest the following criteria for establishing priorities for developing recommendations based on WHO's aims and strategic advantages:

• Problems associated with a high burden of illness in low and middle-income countries, or new and emerging diseases.

• No existing recommendations of good quality.

• The feasibility of developing recommendations that will improve health outcomes, reduce inequities or reduce unnecessary costs if they are implemented.

• Implementation is feasible, will not exhaustively use available resources, and barriers to change are not likely to be so high that they cannot be overcome.

• Additional priorities for WHO include interventions that will likely require system changes and interventions where there might be a conflict in choices between individual and societal perspectives.

**What processes should be used to agree on priorities?:**

• The allocation of resources to the development of recommendations should be part of the routine budgeting process rather than a separate exercise.

• Criteria for establishing priorities should be applied using a systematic and transparent process.

• Because data to inform judgements are often lacking, unmeasured factors should also be considered – explicitly and transparently.

• The process should include consultation with potential end users and other stakeholders, including the public, using well-constructed questions, and possibly using Delphi-like procedures.

• Groups that include stakeholders and people with relevant types of expertise should make decisions. Group processes should ensure full participation by all members of the group.

• The process used to select topics should be documented and open to inspection.

**Should WHO have a centralised or decentralised process?:**

• Both centralised and decentralised processes should be used. Decentralised processes can be considered as separate "tracks".

• Separate tracks should be used for considering issues for specific areas, populations, conditions or concerns. The rationales for designating special tracks should be defined clearly; i.e. why they warrant special consideration.

• Updating of guidelines could also be considered as a separate "track", taking account of issues such as the need for corrections and the availability of new evidence.

## Background

The World Health Organization (WHO), like many other organisations around the world, has recognised the need to use more rigorous processes to ensure that health care recommendations are informed by the best available research evidence. This is the second of a series of 16 reviews that have been prepared as background for advice from the WHO Advisory Committee on Health Research to WHO on how to achieve this. In this paper we address the following questions:

• What criteria should be used to establish priorities?

• What processes should be used to agree on priorities?

• Should WHO have a centralised or decentralised process?

Questions related to group processes for committees developing guidelines and recommendations and priority setting for systematic reviews are addressed in other papers in this series [1,2].

## What WHO is doing now

WHO does not have a centralised process specifically for setting priorities for the development of recommendations. A report of the Director-General to the Executive Board on WHO's strategic budgeting and planning process had this to say about priority setting in general (without specific reference to priorities for recommendations):

*Specific global priorities were included in the procedural guidance for 2002–2003, and measures were provided to ensure a shift of resources to those areas. With regard to regional and country health issues, the team received diverging views. Some staff expressed concern about the little room for specific regional or country priorities, which would not relate directly to the global priorities. Others expressed the need for flexibility during the operational planning phase, which should contribute to the achievement of global priorities in terms of reducing a health problem or improving the health status of the population. It is important to create a monitoring and evaluation system, in which such flexibility can be taken into account*.

The report offered these two recommendations regarding priority setting in general:

*Criteria and parameters for rationalizing the setting of programme priorities should be re-examined with the view to achieving more objectivity*.

*Exercises to shift resources to priority areas should be an integral part of the programme budgeting process, and not taken up as a separate exercise*.

The Guidelines for WHO Guidelines recommends the following [3]:

Guideline development is a process which consumes resources (see Sec VII). They could be developed on almost every health topic or intervention so it is necessary for WHO to decide which topics should be given priority. It is suggested that the following areas be given priority:

*interventions that will require system changes (feasibility concerns) as opposed to those dealing solely with provider/patient interactions. WHO has greater comparative advantage in dealing with governments, for interventions which require inputs and coordination at different levels of the system. It has less comparative advantage on purely provider/patient interactions*.

*cost-effective interventions that address a disease burden which is still causing major health losses, implying under-utilization of the technology (population perspective)*.

*interventions that are of limited or questionable effectiveness but are being used widely (opportunity costs)*.

*Interventions for diseases which have a high burden in developing countries, or new and emerging diseases for which there are no existing guidelines*.

*interventions where there might possibly be a conflict in choices between individual and societal perspectives (political concerns: when countries will need WHO's normative support to make recommendations based on the population perspective especially in the context of other influential organizations espousing guidelines adopting an individual perspective)*.

The Health Evidence Network (HEN), based at the European Regional Office of WHO [4], *collects policy concerns and questions from several sources and through both a proactive and a reactive approach:*

Proactive:

1. Call for topics once a year, through a simple and user-friendly questionnaire to Ministries of Health of Members States, WHO technical units (TUs) including European observatory for Health care systems

2. Reviewing the work already done by HEN Members as well as their work in progress

3. Review of minutes of EU' Parliament

Reactive:

1. The Health Evidence Network To the HEN e-mailbox or direct requests from policymakers

*2. Specific questions or policy concerns identified by the Observatory or WHO Technical Units in their processes of production of papers*.

*Once collected, all this information is translated by the HEN team into answerable questions. The list of questions is then presented to the Steering Committee once a year for its prioritization according to policy relevance, feasibility, timelines, controversy, existing evidence*.

## What other organisations are doing

About 30% of respondents to an international survey of producers of clinical practice guidelines or health technology assessments reported using explicit methods of setting priorities, including the use of explicit criteria, formal consensus processes, and burden of disease [4]. The US Community Preventive Services Task Force, for example,

*chooses broad topics (e.g., tobacco use, cancer, diabetes, the social environment) for review on the basis of the public health burden of the problem; how preventable it is; how it relates to other public health initiatives; and the current level of research and practice activity in public health, clinical, and other settings. The agenda-setting process incorporates input from interested others*.

*The process of selecting specific interventions for review within those topics involves developing a candidate list of interventions, and setting priorities using a voting procedure among the team and the consultants. The Task Force approves or modifies the resulting priorities. Priority-setting criteria that are adapted for the reviews include perceived potential to reduce the burden of disease and injury; potential to increase healthy behaviors and reduce unhealthy behaviors; potential to increase the implementation of effective but not widely used interventions; potential to phase out widely used, less effective interventions in favor of more effective or more cost-effective options; and current level of interest among providers and decision makers. Other priority-setting criteria may be added as relevant and appropriate. Occasionally, review teams have engaged in formal scoring and weighting of the criteria. One or more rounds of this process results in a prioritized list of interventions*.

Other respondents to the survey reported selecting topics based on consultations with their constituencies, requests from end-users, or decisions made by expert panels or a steering group without explicit methods. Seventeen of 67 respondents (25%) reported involving target users in the groups that set priorities.

## Methods

The methods used to prepare this review are described in the introduction to this series [5]. Briefly, the key questions addressed in this paper were vetted amongst the authors and the ACHR Subcommittee on the Use of Research Evidence (SURE). We did not conduct a full systematic review. We searched PubMed and three databases of methodological studies (the Cochrane Methodology Register [6], the US National Guideline Clearinghouse [7], and the Guidelines International Network [8]) for existing systematic reviews and relevant methodological research that address these questions. The answers to the questions are our conclusions based on the available evidence, consideration of what WHO and other organisations are doing, and logical arguments.

For this review we searched PubMed using (clinical practice guidelines or public health guidelines) and (priority setting or setting priorities) and related articles for selected references [9,10]. We searched the Cochrane Methodology Register using priority or priorities. We reviewed the website of the 5^th ^International Conference on Priorities in Health Care [11] and references that we had in our files [12-15].

## Findings

### What criteria should be used to establish priorities?

The US Institute of Medicine's (IOM) Committee on Methods for Setting Priorities for Guidelines Development in its study of setting priorities for clinical practice guidelines published in 1995 argued that the priority setting process should be open and defensible [14] They recommended six general criteria: prevalence, burden of illness, cost of managing the problem, variability in practice, potential of a guideline to improve health outcomes, and potential of a guideline to reduce costs. Because data used to make these judgements is often lacking, they suggested explicit opportunities for important unmeasured factors to be considered. They further suggested separate "tracks" for considering issues for specific populations, conditions or concerns. They argued that the rationales for designating special tracks should be defined clearly; i.e. why they warrant special consideration. They suggested that updating of guidelines should also be considered as a separate "track", taking account of issues such as the need for corrections and the availability of new evidence.

Oortwijn identified 25 criteria used to prioritise health technology assessments and categorised these into four broad categories: burden of disease, potential effects, potential costs, and uncertainty regarding application of the technology [15].

In a more recent selective review for the New Zealand Guidelines Group, the following criteria were identified as indicating that a topic is suitable for guideline development [16]:

*1. The topic is clinically important affecting large numbers of people with substantial morbidity or mortality (the burden of illness)*.

*2. The topic is complex enough to initiate debate about the recommendations*.

*3. There is evidence of variation between actual and appropriate care*.

*4. There are no existing valid guidelines available to use*.

*5. There is an adequate amount of existing evidence available*.

*6. The recommendations will be acceptable to the potential users*.

*7. Implementation of the guideline is feasible, will not exhaustively use the communities' resources, and barriers to clinical change are not so high that they cannot be overcome*.

While burden of disease is commonly used as a criterion for priority setting, it should be noted that the use of summary burden of disease measures, such as disability adjusted life years (DALYs) has been criticised for focusing on disease rather than resource use and interventions, because of the assumptions about values inherent in such measures, and because of the technical limitations of such measures (see for example references [17] and [18]).

### What processes should be used to agree on priorities?

Batista and Hodge in a review conducted 10 years ago found only three articles pertinent to priority setting for clinical practice guidelines [10]. They suggested the following framework for priority setting:

1. Consult with end users and other stakeholders before selecting topics.

2. Consider feasibility during the consultation.

3. Document the process used to select guideline topics.

The IOM suggested the following procedures [14]:

• the use of Delphi-like procedures for obtaining expert judgments or topic rankings through correspondence

• the use of questions that are specific, explicit and consistent with standard methods for questionnaire construction

• experimentation with more formal procedures to arrive at group judgments

They also suggested there is a need to define more narrowly and precisely topics for guideline development. They argued that this would result in more efficient organization of panels and their work, resolution of some apparent controversies, more responsive guidelines, and easier implementation.

Oortwijn identified six steps in the development of practical procedures for setting priorities [15]:

1. Clarifying goals and responsibilities:

*2. Choosing a general approach, method, and criteria for prioritisation*;

*3. Establishing advisory mechanisms and relations with external bodies*;

*4. Establishing arrangements to support and manage the procedure*;

5. Defining a time table and cycle of activity; and

*6. Evaluating and developing the procedure*.

She further identified the following ways in which approaches to priority setting can vary:

• *the extent to which the procedure is explicit and systematic*

• *the extent to which external input and advice is accepted or actively sought*

• *the relative weight given to the views of decision-makers, researchers, and others*

• *the extent to which the procedure is transparent*

• *the effort and resources devoted to the procedure*

Her main conclusion was that explicit and transparent priority setting for health technology assessment is feasible, but that some important methodological issues need to be addressed to ensure that the procedure used is valid, reliable, consistent and useful for policy making.

There is some debate, variation in practice, and limited data regarding involvement of the public in priority setting. There is limited evidence from a small survey in Australia that the public overwhelmingly want their preferences to inform priority-setting decisions [19].

### Should WHO have a centralised or decentralised process?

There are two ways in which priority setting is currently decentralised: geographically (across headquarters, regional offices and countries), and across technical departments. There are limited findings in the literature to inform decisions about how this might best be handled. The IOM noted, "that it is unreasonable – indeed impossible – to expect nationally developed guidelines to cover every operational issue for every kind of setting". "Yet guidelines that leave too much to be decided at the local level or during implementation run the risk of being ignored, misused, and modified in ways detrimental to patients." This is even more so for internationally developed guidelines. Priority setting at each level should draw on the strengths and minimize the limitations of international, national and local organizations. Thus, both centralised and decentralised processes that take account of these different strengths and limitations, as well as needs, are necessary.

## Discussion

WHO has limited resources and limited technical capacity for developing recommendations. It is essential that it should set priorities for how best to use the resources and capacity it has. We did not find an empirical basis for deciding how best to set priorities. However, the use of explicit criteria and systematic processes are more likely than implicit criteria and non-systematic processes to ensure open and defensible priority setting. Based on the experience of other organisations, logic and the aims and strategic advantages of WHO we suggest that the following criteria should be used to set priorities:

• Problems associated with a high burden of illness in low and middle-income countries, or new and emerging diseases.

• No existing guidelines or recommendations of good quality.

• The feasibility of developing recommendations that will improve health outcomes, reduce inequities or reduce unnecessary costs if they are implemented.

• Implementation is feasible, will not exhaustively use available resources, and barriers to change are not likely to be so high that they cannot be overcome.

• Additional priorities for WHO include interventions that will likely require system changes and interventions where there might be a conflict in choices between individual and societal perspectives.

The application of these criteria requires judgements. Appropriate processes are needed, in addition to explicit criteria, to ensure that these judgements are made openly, that they are taken account of in how WHO uses its resources, and that they reflect the priorities of WHO's member states, particularly those of low and middle-income countries. We suggest that the following processes be used for these reasons:

• The allocation of resources to the development of recommendations should be part of the routine budgeting process rather than a separate exercise.

• Criteria for establishing priorities should be applied using a systematic and transparent process.

• Because data to inform judgements are often lacking, unmeasured factors should also be considered – explicitly and transparently.

• The process should include consultation with potential end users and other stakeholders, including the public, using well-constructed questions, and possibly using Delphi-like procedures.

• Groups that include stakeholders and people with relevant types of expertise should make decisions. Group processes should ensure full participation by all members of the group.

• The process used to select topics should be documented and open to inspection.

Both centralised and decentralised processes should be used to take account of different strengths, limitations and needs within WHO across headquarters, regions and countries; and across different technical areas. Drawing on the suggestion of the IOM for having different tracks for considering issues for specific populations, conditions or concerns [14], we suggest:

• Both centralised and decentralised processes should be used. Decentralised processes can be considered as separate "tracks".

• Separate tracks should be used for considering issues for specific areas, populations, conditions or concerns. The rationales for designating special tracks should be defined clearly; i.e. why they warrant special consideration.

• Updating of guidelines could also be considered as a separate "track", taking account of issues such as the need for corrections and the availability of new evidence.

## Further work

Many organisations are now using explicit and systematic priority setting processes for practice guidelines and health technology assessments. A more comprehensive and systematic survey of this experience could inform decisions about processes WHO should use to set priorities for recommendations. Because there is uncertainty about the best ways to set priorities, the processes that are used should be evaluated. When feasible and relevant, alternative processes should be directly compared with respect to the priorities that are generated and the resources that are used.

## Competing interests

ADO and AF work for the Norwegian Knowledge Centre forthe Health Services, an agency funded by the Norwegian government that produces systematic reviews and health technology assessments. All three authors are contributors to the Cochrane Collaboration. ADO and HJS are members of the GRADE Working Group. HJS is documents editor and chair of the documents development and implementation committee for the American Thoracic Society and senior editor of the American College of Chest Physicians' Antithrombotic and Thrombolytic Therapy Guidelines.

## Authors' contributions

ADO prepared the first draft of this review. HJS and AF contributed to drafting and revising it.
